# Impact of Nutritional and Organoleptic Use of Underutilized Green Leafy Vegetables in Bakery Products: A Systematic Review of Novel Food Applications.

**DOI:** 10.12688/f1000research.180136.1

**Published:** 2026-05-14

**Authors:** Madhusudhanan Santhanam, Somu Ga, Senthil Kumaran Piramanayagam, Suresh Sukumar, Nagarajan Theruveethi, Anusha Priyadarsini Kumaresan, Nishar Ahmed Jalaludeen, Rajmohan Dhandapani, Guruprasad Vijayasarathi

**Affiliations:** 1 Department of Culinary Arts,Welcomgroup Graduate School of Hotel Administration, Manipal Academy of Higher Education, Manipal, Karnataka, 576104, India; 2 Department of Hospital Administration, Kasturba Medical College,, Manipal Academy of Higher Education, Manipal, Karnataka, 576104, India; 3 Department of Allied Hospitality Studies, Welcomgroup Graduate School of Hotel Administration, Manipal Academy of Higher Education, Manipal, Karnataka, 576104, India; 4 Department of Medical Imaging Technology, Manipal College of Health Professions, Manipal Academy of Higher Education, Manipal, Karnataka, 576104, India; 5 Department of Optometry. Manipal College of Health Professions, Manipal Academy of Higher Education, Manipal, Karnataka, 576104, India; 6 Department of Dietetics and Applied Nutrition, Welcomgroup Graduate School of Hotel Administration, Manipal Academy of Higher Education, Manipal, Karnataka, 576104, India; 7 Department of Food and Beverage Service, Welcomgroup Graduate School of Hotel Administration, Manipal Academy of Higher Education, Manipal, Karnataka, 576104, India; 8 Department of Culinary Arts, Welcomgroup Graduate School of Hotel Administration, Manipal Academy of Higher Education, Manipal, Karnataka, 576104, India; 9 School of Rehabilitation and Medical Sciences, College of Health Sciences, University of Nizwa, Nizwa,Sultanate of, Oman

**Keywords:** Underutilized Green Leafy Vegetables (GLVs), Bakery Product Fortification, Nutritional Enhancement, Sensory Acceptability, Functional Food Applications.

## Abstract

**Background:**

The low utilization of green leafy vegetables (GLVs) makes them a nutrient-dense food that is frequently overlooked in the food system. Their integration into baked products may confer nutritional and organizational benefits. However, few studies have addressed their impact on bakery formulations.

**Objectives:**

This systematic review aimed to jointly appraise the nutritional enrichment and sensory properties of bakery products enhanced with the incorporation of underutilized GLVs, and to highlight their potential new food and industrial applications.

**Methods:**

Systematic searches were conducted in scientific databases, PubMed, Scopus, Google Scholar, and Web of Science, according to the PRISMA guidelines. Only primary peer-reviewed journal articles that focused on assessing the nutritional profile, sensory attributes, and technological feasibility of incorporating GLVs into bakery products were selected for this review. We documented the micronutrient content, bioactive components, consumer acceptability, and technological challenges.

**Results:**

One significant difference found in food products was that GLV incorporation increased essential nutrients. Bioactive compounds from GLVs offer health benefits including improved glycemic control and reduced oxidative stress. However, sensory acceptability remains an issue, with bitterness and texture as main obstacles. Consumer studies show acceptable GLV levels depend on species and processing methods. Techniques such as dehydration and fermentation mitigate unwanted sensory effects.

**Conclusions:**

As we incorporate more underutilised green leafy vegetables in bakery products, it becomes increasingly evident that they have substantial potential to provide significant nutritional and functional benefits to humans. Future research should address the fine-tuning of formulations to maximize benefits while meeting consumer preferences and ensuring commercial feasibility on a large scale in the food industry.

**Registration:**

This systematic review was registered with the Open Science Framework (OSF). DOI: https://doi.org/10.17605/OSF.IO/7YHDZ.

## Introduction

### Underutilised green leafy vegetable (uglv): an overview

UGLVs are a diverse group of plants that have been historically overlooked in mainstream agriculture but are now gaining attention for their nutritional and medicinal properties. These vegetables are rich in essential nutrients and bioactive compounds, making them valuable for enhancing dietary diversity and addressing nutritional deficiencies in the diet. Many green, leafy vegetables are important for rural folk, as they are a major part of their diet and nutrition. These vegetables, once held in high esteem for their nutritional and medicinal benefits, are now underutilized and unexplored due to changing dietary preferences, lack of awareness, and modern agricultural practices favouring commercially popular crops. The present study aimed to compare the growth and yield performance of different underutilized green leafy vegetables. The growth performance analysis revealed the maximum plant height, leaf breadth, number of leaves per plant, leaf length, leaf area, and yield in different vegetable varieties. Centella asiatica (L.) Urb. is an important medicinal herb and a nutraceutical herb that accumulates highly pentacyclic triterpenoid saponins, an active compound that induces cell rejuvenation to promote physical and mental health ( Bhavithra et al., 2019). Other purposes for which Centella asiatica is used include being a green leafy vegetable for juice, drinks, and food preparation. Ipomoea aquatica Forsk. is a common green leafy vegetable grown throughout Southeast Asia. It is a rich source of vitamins, minerals, proteins, fiber, carotenoids, and flavonoids, which bestow various health benefits ( Joshi & Chaturvedi, 2013). Although it is a common weed in lakes and freshwater ponds, it is often overlooked because of the lack of knowledge about this green leafy vegetable. Chench (Corchorus acutangulus Lam.) is an unexploited and underutilized leafy vegetable in India ( Kurrey et al., 2018). It is a good source of nutrients, including water, energy, protein, fat, carbohydrates, fiber, calcium, phosphorus, iron, and ascorbic acid. The yummiest bitterness in Corchorus leaves is due to the presence of Corchorin glycosides. The growth of green leafy vegetables is highly dependent on several influencing contextual factors, such as the locality's environmental features, climatic conditions, and agronomic activities conducted in that area ( Kurrey et al., 2018). A concept or logic model should be designed for the digital information management of green leafy vegetable production, allowing for the implementation of information management systems, including the safety monitoring of green leafy vegetables through barcodes ( Shen et al., 2007).

Bakery products can be markedly improved in their mineral and fiber contents by using green leafy vegetables such as spinach, chard, and kale. For example, the mineral content has been enhanced by spinach and chard, while the fiber content has been enhanced with other vegetables, such as onions and pak choi ( Santamaria et al., 2023). Various studies have focused on developing value-added products using these underutilized leaves ( Joshi & Mathur, 2015; Khatoniar et al., 2018). Although the integration of these underutilized grains and their ingredients shows great promise, consumers must still accept these products, and consistency in product quality must be assured. Therefore, further research should focus on the formulation and processing of novel bakery solutions.

## Methodology

A systematic literature review was conducted to evaluate the nutritional and organoleptic applications of underutilized green leafy vegetables (GLVs) in bakery products. Four major databases were used to search the literature: Web of Science, Scopus, PubMed, and Google Scholar. There were no language restrictions on this search to ensure a thorough review of the published papers.

The search strategy involved different combinations of Medical Subject Headings (MeSH) terms to optimize the search results for relevant studies. The search terms include “impact” OR “nutritional use” AND “organoleptic use” AND “nutritional and organoleptic use” AND “impact on nutritional and organoleptic use” AND “underutilized green leafy vegetables” AND “GLV” AND “use of GLV in bakery products.” These terms were then combined in various combinations to identify research studies in which GLVs were incorporated into bakery formulations, and their effects on nutritional composition, sensory attributes, and consumer acceptance were evaluated.

To guarantee the reliability and transparency of the review process, the protocol of this study was registered in the Open Science Framework (OSF); DOI: https://doi.org/10.17605/OSF.IO/7YHDZ ( SANTHANAM et al., 2026c). This review follows the Preferred Reporting Items for Systematic Reviews and Meta-Analyses (PRISMA) 2020 and PRISMA 2020 for abstract checklists, which can be accessed on the open-access repository: Figshare at https://doi.org/10.6084/m9.figshare.31878151 ( Santhanam et al., 2026e). The table below contains further details on the search strategies, including database-specific queries ( Table 1) and inclusion criteria and exclusion criteria ( Table 2).

**Table 1.** Overview of the search strategy and the count of publications across each electronic database.

Database	Search Strategy	No.of Publications
PubMed	Review”[TITLE/ABSTRACT]) AND (“Food Application”[TITLE/ABSTRACT]) AND (“Novel Food Application”[TITLE/ABSTRACT])	1706
Scopus	(“Underutilized green leafy vegetables”) AND TITLE-ABS-KEY (“GLV”) AND TITLE-ABS-KEY (“Impact on nutritional and organoleptic use”) AND TITLE-ABS-KEY (“Use of GLV in bakery products”) OR TITLE-ABS-KEY (“Food Application”) AND TITLE-ABS-KEY (“Novel Food Application”)	580
Web of Science	TS = (“Nutritional use” OR “Organoleptic use” OR “Nutritional and organoleptic use” OR “Impact on nutritional and organoleptic use”) AND TS = (“Underutilized green leafy vegetables” OR “GLV”) AND “Use of GLV in bakery products”) AND (“Novel food application”)	1109
Google Scholar	Allintitle (“Underutilized green leafy vegetables” OR “GLV”) AND (“Impact on nutritional and organoleptic use”) AND (“Use of GLV in bakery products”) AND (“Food application”) AND (“Novel food application”) AND (“Nutritional enhancement”) AND (“Sensory Acceptability) AND (“Fortification”)	5024

**Table 2.** Criteria for inclusion and exclusion of studies.

Inclusion criteria	Exclusion criteria
Review articles, meta-analyses, and systematic reviews relevant to the topic.	Studies lacking experimental data or proper control groups.
Studies that evaluate the nutritional and organoleptic properties of underutilized green leafy vegetables (GLVs).	Research not specifically focused on underutilized GLVs in bakery products.
Studies examining the impact of GLVs on the nutritional profile, sensory attributes (color, texture, taste, aroma), and functional properties of bakery products.	Studies assessing consumer acceptability, sensory evaluation scores, and shelf-life improvement.
Reports on changes in nutritional composition (protein, fiber, vitamins, minerals, bioactive compounds) in bakery products with GLVs.	Studies not reporting nutritional analysis or sensory evaluation results.
Last ten years articles were included for conducting systematic review.	Articles published before ten years are excluded.

## Results

**Figure 1.**
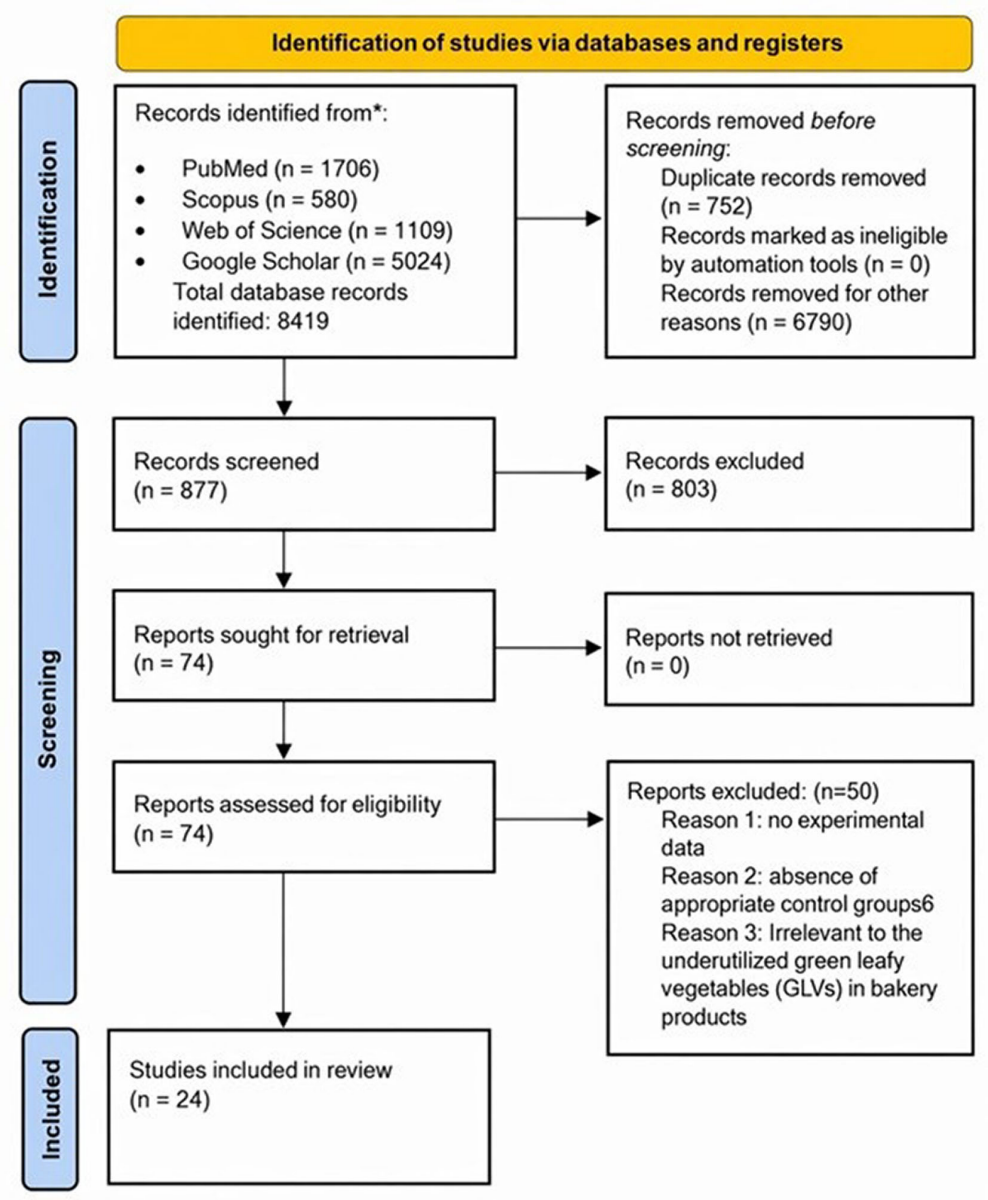
PRISMA 2020 flow diagram illustrating the review process. (n: total count).

The current systematic review follows the Preferred Reporting Items for Systematic Reviews and Meta-Analyses (PRISMA) statement in reporting for systematic reviews. The study selection process is illustrated in the PRISMA flow diagram ( Figure 1). A search of four electronic databases (PubMed, Scopus, Web of Science, and Google Scholar) yielded a total of 8419 studies. After removing 752 duplicate records, 877 articles were subjected to title and abstract screening. Seventy-four full-text articles were assessed for eligibility, and 50 were excluded because of the lack of experimental data, absence of appropriate control groups, or irrelevance to underutilized green leafy vegetables (GLVs) in bakery products. Finally, 24 studies were included in the review ( Gupta et al., 2005; Sarkar et al., 2023; Okunlola et al., 2023; Yadav & Singh, 2024; Bhavithra et al., 2019; Marappan et al., 2024; Pandiyaraj et al., 2024; Joshi & Mathur, 2015; Coppock et al., 1952). PRISMA 2020 flow diagram for systematic review is referred to provide the article screening process ( Figure 1).

### Attributes of included studies

The selection criteria are summarized in Table 3. Of the 24 selected studies, 15 were published in the last ten years and five were studies examining the impact of GLVs on the nutritional profile and sensory attributes, and four evaluated the nutritional and organoleptic properties of underutilized green leafy vegetables. The studies were conducted in several countries, including India, China, the USA, and other European countries, and were published between 2005 and 2025 ( Gupta et al., 2005; Sarkar et al., 2023).

**Table 3.** Features of GLV incorporation in bakery products.

Sr. No.	Sub- Heading	Content to be Discussed	First Author	Year
1	Underutilized GLVs	Underutilized green leafy vegetables (GLVs) are often neglected due to their perception as weeds or inferior plants. However, they possess significant health benefits and can be effectively utilized for food fortification.	Gupta et al.	2005
2	Nutritional Composition	Underutilized GLVs are rich in essential nutrients, including minerals (iron, calcium, zinc), vitamins (beta-carotene, vitamin C, and E), and phytochemicals such as dietary fiber, phenols, and antioxidants.	Sarkar et al.	2023
3	Health Benefits and Medicinal Properties	GLVs contain bioactive compounds that contribute to their medicinal properties, supporting nutritional requirements and providing protection against diseases associated with malnutrition.	Okunlola et al.	2023
4	Food Fortification Potential	Underutilized GLVs have strong potential as food fortificants due to their rich nutritional and bioactive profiles. Processing methods play a crucial role in maintaining their nutritional quality for commercial applications.	Yadav & Singh	2024
5	Challenges and Future Directions	Underutilized GLVs have strong potential as food fortificants due to their rich nutritional and bioactive profiles. Processing methods play a crucial role in maintaining their nutritional quality for commercial applications.	Bhavithra et al.	2019
6	Nutritional Value	GLVs are high in vitamins A, C, and E, as well as important minerals such as potassium and calcium. Some varieties such as cauliflower and carrot leaves have significant protein levels.	Marappan et al.	2024
7	Economic and Agricultural Impact	Cultivation of GLVs can enhance agricultural biodiversity and provide economic benefits to smallholder farmers by reducing dependence on conventional crops.	Pandiyaraj et al.	2024
8	Nutritional Benefits of Underutilized GLVs in Bakery Products	GLVs incorporated into bakery products enhance micronutrient content, including iron, calcium, zinc, and vitamins such as beta-carotene, vitamin E, vitamin K, and vitamin C, contributing to improved health outcomes.	Sarkar et al.	2023
9	Application in Bakery Products	Dehydrated leaf powders from GLVs can be incorporated into bakery products to improve their nutritional profile, particularly through increased micronutrient content.	Joshi & Mathur	2015
10	Organoleptic Properties of Bakery Products Containing GLVs	Sensory attributes such as taste, texture, and overall quality significantly influence consumer acceptance of bakery products. Factors such as flour type, shortening, and additives also affect organoleptic properties.	Coppock et al.	1952

### Attributes of excluded studies

Studies that did not present experimental data were excluded as a criterion by which the merit of the study findings could be judged. Furthermore, studies lacking proper control groups were excluded to allow reliable comparisons between test and control samples. Participants who considered underutilized green leafy vegetables but had no specific inclinations about their application in bakery products were also excluded. Furthermore, studies that did not include any nutritional analysis or sensory evaluation were excluded, as these were essential to show the effect of GLVs on bakery formulations. Articles published before ten years were excluded. There were 50 studies rejected on these grounds.

### Advancement in nutrition bioactive component

The incorporation of underutilized green leafy vegetables (GLVs) into baked goods significantly increased the levels of dietary fiber, vitamins (A, C, E, K, and B-complex), minerals (iron, calcium, and zinc), and bioactive compounds (polyphenols, flavonoids, and carotenoids). Several studies have reported that the addition of GLVs enhances the antioxidant properties of bakery products, thereby contributing to their functional value. For instance, GLV-enriched bakery products exhibited 30–50% higher total phenolic content and antioxidant activity compared to their conventional counterparts ( Sarkar et al., 2023).

**Assessment of Sensory Context and Consumer Perception:** Although improvements in nutritional quality have been observed, sensory attributes such as bitterness, color, and texture remain critical determinants of consumer acceptability. The overall mean acceptability scores across six studies ranged from 6.5 to 8.2 on a 10-point hedonic scale. To address these sensory challenges, various studies have recommended processing techniques such as dehydration and fermentation to reduce undesirable characteristics, as supported by Coppock et al. (1952) and Naqash et al. (2017).

### Challenges of technical feasibility and processing

The major technological challenges associated with the incorporation of underutilized green leafy vegetables (GLVs) into bakery formulations include alterations in dough rheology, texture, and shelf-life stability. The addition of GLV powders influences moisture absorption behavior, thereby affecting dough elasticity and crumb structure. Processing techniques such as fermentation and controlled oxidative treatments can improve texture and palatability. Additionally, drying methods, including hot air drying and freeze drying, are effective in preserving bioactive compounds while maintaining desirable sensory attributes. These technological observations have been reported by Belokurova et al. (2015) and Menaka et al. (2024).

### Study quality evaluation and limitations

Quality assessment based on the Newcastle-Ottawa Scale (NOS) showed that the included studies had scores ranging between 6 and 9, most being medium to high grade. However, heterogeneity in study designs, GLV types, processing methods, and analytic techniques precluded a feasible quantitative synthesis or meta-analysis.

In summary, according to 10 out of 24 studies, the incorporation of GLVs improved the nutritional and functional properties of bakery products, while 14 did not show a significant difference. Owing to the different methodologies and experimental protocols, complete quantitative synthesis is not possible.

## Discussion

Integrating green leafy vegetables (GLVs) into baked goods is a promising strategy to enhance the nutritional value and sustainability of processed foods. Most GLVs have good nutrient compositions, but they remain underutilized because of perishability, sensory difficulties, and lack of consumer knowledge. This discussion will explore facilitation of the incorporation of GLVs into baked products along with the technological, nutritional, and functional aspects of GLVs in bakery products and the challenges and opportunities that arise with them. Advanced technologies, such as ingredient substitution and process optimization, will make GLV incorporation healthy and viable while maintaining the structural integrity of the preferred bakery product. In addition, new applications concerning largely ignored GLVs and traditional grains may resolve sustainability issues in the development of new products. The functional application of these ingredients provides antioxidant capacity and improved product stability. However, emerging barriers to perishability, formulation, and market acceptance should be addressed using innovative processing technologies and consumer education.

The table 3 and 4 present the broad features of GLV incorporation in bakery products, including technological, health, and possible obstacles in upscaling applications. Technical challenges include dough rheology, texture, and shelf-life stability issues. Fermentation and drying processes can improve the texture and maintain the bioactive compounds.

**Table 4.** Features of GLV incorporation in bakery products.

Sr. No.	Sub- Heading	Content to be Discussed	First Author	Year
01	Technological Aspects and Challenges in Incorporating GLVs into Bakery Products	Incorporation of GLVs affects dough rheology, texture, and shelf-life stability. Challenges include interference with fermentation, optimization of processing conditions, and increased production costs. Gluten-free formulations further complicate product development due to the absence of gluten structure.	Belokurova et al.	2015
	Technological Approaches	Approaches include gluten-free flour substitution, process optimization, and fermentation techniques such as sourdough fermentation. Advanced processing methods improve product quality and stability.	Naqash et al., Menaka et al.	2017, 2024
	Nutritional Enhancement	GLVs enhance mineral and fiber content, while vegetable powders contribute antioxidants, vitamins, and bioactive compounds. Functional ingredients and by-products improve sustainability and nutritional quality.	Santamaria et al.; Salehi et al.	2023; 2019
	Challenges in Quality	Issues such as altered texture, volume, and color arise due to gluten absence and incorporation of vegetable flours. Rheological changes affect dough consistency and final product quality.	Alashbayeva et al.; Gallardo et al.	2024
02	Novel Applications of Underutilized GLVs in Bakery Products	Underutilized leaves such as beetroot, carrot, and cauliflower are incorporated to improve nutritional quality. Lactic acid bacteria enhance shelf life and functionality, while fiber concentrates increase antioxidant properties.	Joshi & Mathur; Liu et al.	2015, 2022
	Ancient Grains in Bakery Products	Ancient grains such as spelt and einkorn improve nutritional value but require process optimization. Hydrocolloid use and hydration control enhance baking performance.	Valsalan et al.	2024
	Nutritional Enhancement	Incorporation of GLVs such as broccoli leaves increases antioxidant potential and micronutrient content. Protein and fiber enrichment aligns with consumer health trends.	Krupa-Kozak et al., P. Kumar et al., Cetiner et al.	2021; 2022; 2023
	Functional Properties	GLVs improve functional properties through bioactive compounds, enhanced digestibility, and health-promoting effects such as antioxidant and anti-glycation activity.	Krupa-Kozak et al.	2021
	Sustainability and Waste Reduction	Use of vegetable by-products supports sustainability by reducing food waste. Revalorization techniques enhance resource efficiency in food systems.	Martinez & Gómez; Cano-Lamadrid et al	2024; 2022
03	Health Implications and Functional Benefits of Bakery Products with GLVs	GLVs provide essential nutrients including fiber, vitamins, and minerals, contributing to the prevention of chronic diseases and micronutrient deficiencies. Limited awareness restricts their widespread consumption.		
	Nutritional Benefits	GLVs such as red spinach and amaranth are rich in essential nutrients. Cauliflower leaves contribute significant protein content in bakery formulations.	Sarkar et al., Yadav & Singh	2023; 2024
	Functional Properties	High phenolic content in GLVs helps reduce oxidative stress. Increased dietary fiber improves digestion and satiety.	Sarkar et al.	2023
	Health Implications	Regular consumption of GLVs reduces risks of chronic diseases and supports functional food development due to bioactive compounds.	Sarkar et al.	2023
04	Barriers and Opportunities for Scaling Up Green Leafy Vegetable Use in Bakery Products	Challenges include perishability, changes in dough rheology, and limited consumer acceptance. Opportunities include dehydration, fortification, and innovative product development.	Khatoniar et al.; Liu et al.	2018; 2022
	Barriers to Scaling Up GLV Use	High moisture content leads to spoilage, while changes in dough resistance and extensibility affect product quality.	Naumenko et al.	2024
	Opportunities for Scaling Up GLV Use	Dehydration improves shelf life, while fortification enhances nutritional value. Research-driven product development supports innovation in bakery applications.	Khatoniar et al.; Osipova et al.	2018, 2020

Baked products can be complemented with green leafy vegetables (GLVs) to enhance nutrition and environmental sustainability. GLVs are used sparingly because of their perishability, lack of sensory appeal, and poor consumer awareness. Modern technologies will allow the incorporation of GLVs into products while maintaining their original attributes. Tackling perishability and market acceptability is vital for successful product development.

### Strengths and limitations

This systematic review was conducted according to the PRISMA guidelines. It followed comprehensive and systematic procedures for the selection and analysis of the literature. Research from four major databases (PubMed, Scopus, Web of Science, and Google Scholar) was included in this study, regardless of the language. This negates the possibility of a publication bias. This synthesis evaluates the nutritional and sensory aspects of the improvements brought by incorporating underutilized green leafy vegetables into baked products, thereby functioning as functional foods. The findings will also contribute to the emerging field of sustainable nutrition by positing underutilized GLVs as contributors to greater dietary diversity.

However, this review has some limitations. Most of the studies included were either observational or experimental, making it difficult to establish a direct causation between GLV incorporation and direct health outcomes. Diverse designs, processing methods, and analytic techniques did not permit a meta-analysis in this study. In addition, biases could arise from differences in sensory evaluation methods and consumer acceptability assessments, as these are mainly self-reported. However, it does not investigate the long-term stability or commercial acceptability of GLV-laden bakery products. Future studies must standardize the formulas and employ randomized controlled trials for functional and sensory validation in real-life implementation contexts.

## Conclusions

A systematic review of the effects of underutilized green leafy vegetables (GLVs) in bakery products has drawn several striking conclusions. The nutritional benefits of adding GLVs to baked products include the introduction of vitamins and minerals into baked foods, thereby augmenting dietary diversity and nutritional intake.

GLVs not only improve nutritional value but also contain bioactive compounds that provide health benefits, such as glycemic control and antioxidant effects. Thus, GLVs can be considered potential functional foods that are beneficial for health.

Consumer Sensory Challenges: According to the review, nutritional benefits aside, sensory acceptance continues to be a barrier to consumption. Bitterness and texture can both play a role in consumer acceptance. In addition, the threshold of inclusion of GLVs varies according to the specific GLV and processing methods.

**Technological Solutions:** The review suggests the adoption of technological methods, such as drying and fermentation, to address sensory issues. These techniques will help eliminate unwarranted sensory effects, making the GLV bakery more palatable.

**Need for Standardization:** As observed in this review, future research should be directed toward formulating standardized and randomized controlled trials. In this way, functional and sensory properties can be confirmed in a real-life context, ensuring that consumers are catered to and benefits are maximized.

**Gaps in Knowledge:** The review shows that research shows a wide gap in existing literature, especially regarding studies geared towards establishing direct causa and understanding the activity and contribution of individual GLVs in humans.

## Data Availability

**Figshare**. Data Extraction Table. https://doi.org/10.6084/m9.figshare.31970022 ( Santhanam et al., 2026a). Above file contains the following underlying data: Data extraction table. (The screened articles will be organised in the mentioned data sheet.) **Figshare**. Search Strategy. https://doi.org/10.6084/m9.figshare.31970049 ( Santhanam et al., 2026b). Above file contains the following underlying data: Research study design (The above document includes the study design used for the systematic literature review.) **Open Science Framework (OSF):** Impact of Nutritional and Organoleptic Use of Underutilized Green Leafy Vegetables in Bakery Products: A Systematic Review of Novel Food Applications; DOI: https://doi.org/10.17605/OSF.IO/7YHDZ ( SANTHANAM et al., 2026c). This project contains the following extended data: OSF project files (Systematic review protocol and related documentation). **Figshare**. Data synthesis table. https://doi.org/10.6084/m9.figshare.31970184 ( Santhanam et al., 2026d). This file contains the extended data table of the synthesised articles, which were finalised after screening for eligibility criteria. **Figshare**: PRISMA CHECKLIST AND PRISMA ABSTRACT CHECKLIST; DOI: https://doi.org/10.6084/m9.figshare.31878151 ( Santhanam et al., 2026e). This project contains the following reporting guidelines: PRISMA checklist.pdf (Checklist followed for systematic review reporting). Study data can be accessed according to the terms of the Creative Commons Attribution 4.0 International License (CC-BY 4.0).
