# Impact of Nutritional and Organoleptic Use of Underutilized Green Leafy Vegetables in Bakery Products: A Systematic Review of Novel Food Applications.

**DOI:** 10.12688/f1000research.180136.2

**Published:** 2026-08-26

**Authors:** Madhusudhanan Santhanam, Somu Ga, Senthil Kumaran Piramanayagam, Suresh Sukumar, Nagarajan Theruveethi, Anusha Priyadarsini Kumaresan, Nishar Ahmed Jalaludeen, Rajmohan Dhandapani, Guruprasad Vijayasarathi

**Affiliations:** 1 Department of Culinary Arts,Welcomgroup Graduate School of Hotel Administration, Manipal Academy of Higher Education, Manipal, Karnataka, 576104, India; 2 Department of Hospital Administration, Kasturba Medical College,, Manipal Academy of Higher Education, Manipal, Karnataka, 576104, India; 3 Department of Allied Hospitality Studies, Welcomgroup Graduate School of Hotel Administration, Manipal Academy of Higher Education, Manipal, Karnataka, 576104, India; 4 Department of Medical Imaging Technology, Manipal College of Health Professions, Manipal Academy of Higher Education, Manipal, Karnataka, 576104, India; 5 Department of Optometry. Manipal College of Health Professions, Manipal Academy of Higher Education, Manipal, Karnataka, 576104, India; 6 Department of Dietetics and Applied Nutrition, Welcomgroup Graduate School of Hotel Administration, Manipal Academy of Higher Education, Manipal, Karnataka, 576104, India; 7 Department of Food and Beverage Service, Welcomgroup Graduate School of Hotel Administration, Manipal Academy of Higher Education, Manipal, Karnataka, 576104, India; 8 Department of Culinary Arts, Welcomgroup Graduate School of Hotel Administration, Manipal Academy of Higher Education, Manipal, Karnataka, 576104, India; 9 School of Rehabilitation and Medical Sciences, College of Health Sciences, University of Nizwa, Nizwa,Sultanate of, Oman

**Keywords:** Underutilized Green Leafy Vegetables (GLVs), Bakery Product Fortification, Nutritional Enhancement, Sensory Acceptability, Functional Food Applications.

## Abstract

**Background:**

The low utilization of green leafy vegetables (GLVs) makes them a nutrient-dense food that is frequently overlooked in the food system. Their integration into baked products may confer nutritional and organizational benefits. However, few studies have addressed their impact on bakery formulations.

**Objectives:**

This systematic review aimed to jointly appraise the nutritional enrichment and sensory properties of bakery products enhanced with the incorporation of underutilized GLVs, and to highlight their potential new food and industrial applications.

**Methods:**

Systematic searches were conducted in scientific databases, PubMed, Scopus, Google Scholar, and Web of Science, according to the PRISMA guidelines. Original peer-reviewed research articles reporting nutritional, sensory, functional, technological, or organoleptic outcomes associated with the incorporation of underutilized green leafy vegetables into bakery products. We documented the micronutrient content, bioactive components, consumer acceptability, and technological challenges.

**Results:**

One significant difference found in food products was that GLV incorporation increased essential nutrients. Bioactive compounds from GLVs offer health benefits including improved glycemic control and reduced oxidative stress. However, sensory acceptability remains an issue, with bitterness and texture as main obstacles. Consumer studies show acceptable GLV levels depend on species and processing methods. Techniques such as dehydration and fermentation mitigate unwanted sensory effects.

**Conclusions:**

As we incorporate more underutilised green leafy vegetables in bakery products, it becomes increasingly evident that they have substantial potential to provide significant nutritional and functional benefits to humans. Future research should address the fine-tuning of formulations to maximize benefits while meeting consumer preferences and ensuring commercial feasibility on a large scale in the food industry.

**Registration:**

This systematic review was registered with the Open Science Framework (OSF). DOI: https://doi.org/10.17605/OSF.IO/7YHDZ.

## Introduction

### Underutilised green leafy vegetable (UGLV): an overview

UGLVs are a diverse group of plants that have been historically overlooked in mainstream agriculture but are now gaining attention for their nutritional and medicinal properties. These vegetables are rich in essential nutrients and bioactive compounds, making them valuable for enhancing dietary diversity and addressing nutritional deficiencies in the diet. Many green, leafy vegetables are important for rural folk, as they are a major part of their diet and nutrition. These vegetables, once held in high esteem for their nutritional and medicinal benefits, are now underutilized and unexplored due to changing dietary preferences, lack of awareness, and modern agricultural practices favouring commercially popular crops. The present study aimed to compare the growth and yield performance of different underutilized green leafy vegetables. The growth performance analysis revealed the maximum plant height, leaf breadth, number of leaves per plant, leaf length, leaf area, and yield in different vegetable varieties. Centella asiatica (L.) Urb. is an important medicinal herb and a nutraceutical herb that accumulates highly pentacyclic triterpenoid saponins, an active compound that induces cell rejuvenation to promote physical and mental health ( Bhavithra et al., 2019). Other purposes for which Centella asiatica is used include being a green leafy vegetable for juice, drinks, and food preparation. Ipomoea aquatica Forsk. is a common green leafy vegetable grown throughout Southeast Asia. It is a rich source of vitamins, minerals, proteins, fiber, carotenoids, and flavonoids, which bestow various health benefits ( Joshi & Chaturvedi, 2013). Although it is a common weed in lakes and freshwater ponds, it is often overlooked because of the lack of knowledge about this green leafy vegetable. Chench (Corchorus acutangulus Lam.) is an unexploited and underutilized leafy vegetable in India ( Kurrey et al., 2018). It is a good source of nutrients, including water, energy, protein, fat, carbohydrates, fiber, calcium, phosphorus, iron, and ascorbic acid. The yummiest bitterness in Corchorus leaves is due to the presence of Corchorin glycosides. The growth of green leafy vegetables is highly dependent on several influencing contextual factors, such as the locality's environmental features, climatic conditions, and agronomic activities conducted in that area ( Kurrey et al., 2018). A concept or logic model should be designed for the digital information management of green leafy vegetable production, allowing for the implementation of information management systems, including the safety monitoring of green leafy vegetables through barcodes ( Shen et al., 2007).

Micronutrient deficiencies, commonly referred to as hidden hunger, remain a major global public health challenge, particularly in low- and middle-income countries, where deficiencies of iron, vitamin A, zinc, and calcium contribute to anaemia, impaired immunity, poor cognitive development, and increased disease burden. Addressing these deficiencies requires sustainable dietary diversification rather than reliance solely on supplementation or conventional food fortification. In this context, underutilized green leafy vegetables (UGLVs) are gaining recognition as nutrient-dense, affordable, and locally available food resources rich in vitamins, minerals, dietary fibre, and bioactive phytochemicals. Their utilization aligns with the United Nations Sustainable Development Goal 2 (Zero Hunger) by promoting nutritional security, agricultural biodiversity, and sustainable food systems. Concurrently, the growing demand for functional foods has encouraged the incorporation of nutrient-rich plant ingredients into widely consumed food products. Bakery products, including bread, biscuits, cookies, cakes, crackers, and muffins, are considered ideal vehicles for nutritional fortification because of their widespread consumption, consumer acceptance, and potential to deliver health-promoting ingredients. Consequently, incorporating UGLVs into bakery formulations represents a promising strategy to enhance nutritional quality, diversify diets, and develop functional foods while utilizing underexploited plant resources.

Bakery products can be markedly improved in their mineral and fiber contents by using green leafy vegetables such as spinach, chard, and kale. For example, the mineral content has been enhanced by spinach and chard, while the fiber content has been enhanced with other vegetables, such as onions and pak choi ( Santamaria et al., 2023). Various studies have focused on developing value-added products using these underutilized leaves ( Joshi & Mathur, 2015; Khatoniar et al., 2018). Although the integration of these underutilized grains and their ingredients shows great promise, consumers must still accept these products, and consistency in product quality must be assured. Therefore, further research should focus on the formulation and processing of novel bakery solutions.

Although several studies have evaluated individual green leafy vegetables in specific bakery products, the available evidence remains fragmented across vegetable species, processing methods, bakery matrices, and sensory outcomes. No comprehensive synthesis has simultaneously evaluated nutritional enhancement, organoleptic performance, technological feasibility, and commercial implications of underutilized green leafy vegetables in bakery applications. This systematic review addresses the following knowledge gaps.

### Research gap

• Lack of standardized GLV incorporation levels
• Few comparisons across bakery products
• Limited sensory synthesis
• No evaluation of commercial feasibility
• No comparison of processing methods
• Limited discussion on nutrient retention

### Operational definition and classification of Underutilized Green Leafy Vegetables (UGLVs)

For the purpose of this systematic review, Underutilized Green Leafy Vegetables (UGLVs) are taken to be edible leafy plant species that have meaningful nutritional, functional, and medicinal features, yet they are still not really used enough in mainstream farming production, commercial food chains and everyday consumer eating patterns. Even though it is often said they can support nutritional security, help with diet variety, and strengthen sustainable food systems, in practice these vegetables still end up getting less scientific focus, fewer cultivation pushes, and lower market visibility than the leafy vegetables people already eat in a regular way ( Gupta et al., 2005; Bhavithra et al., 2019; Sarkar et al., 2023).

The way UGLVs were sorted in this review followed the traits that are usually described in the literature about underutilized and neglected vegetable crops. A vegetable was treated as underutilized if it matched one or more of these points: (i) small scale commercial cultivation and overall production; (ii) poor market presence and weak consumer awareness; (iii) a narrow geographical spread, or use that stays within traditional, indigenous, or very local food systems; (iv) nutritional, functional, or medicinal advantages that have backing from scientific results; and (v) the possibility to aid food and nutrition security, agricultural biodiversity, and more sustainable dietary routines ( Gupta et al., 2005; Kanwar et al., 2022; Sarkar et al., 2023).

Accordingly, vegetable species in Table 1 such as Centella asiatica (Indian pennywort), Ipomoea aquatica (water spinach), Corchorus acutangulus (jute mallow/chench), Basella alba (Malabar spinach), Amaranthus spp. (amaranth greens), and other leafy vegetables that are regionally consumed and mentioned in the included studies, were treated as UGLVs within the boundary of this review ( Bhavithra et al., 2019; Kumar et al., 2022; Sarkar et al., 2023). Using this operational definition helped keep things consistent across study selection, data pulling, and evidence synthesis, and it also supported a sharper look at how UGLVs could be used for nutritional and organoleptic purposes in bakery products.

**Table 1.** Classification of underutilized green leafy vegetables included in the review.

Common name	Scientific name	Family	Region of traditional use	Reason for classification as UGLV
Indian Pennywort	*Centella asiatica*	Apiaceae	India, Sri Lanka	Limited commercial cultivation, high nutraceutical value
Water Spinach	*Ipomoea aquatica*	Convolvulaceae	South and Southeast Asia	Regional consumption, rich micronutrient profile
Chench (Jute Mallow)	*Corchorus acutangulus*	Malvaceae	India	Underexploited crop with high nutritional value
Malabar Spinach	*Basella alba*	Basellaceae	India, Asia	Traditional use but limited industrial utilization
Amaranth Greens	*Amaranthus spp.*	Amaranthaceae	Asia and Africa	High nutrient density, low commercial penetration

Source: Adapted from Gupta et al. (2005), Bhavithra et al. (2019), Kanwar et al. (2022), Kumar et al. (2022), and Sarkar et al. (2023).

## Methodology

A systematic literature search was done through PubMed, Scopus, Web of Science, and Google Scholar, to find studies that actually assessed nutritional and organoleptic applications of underutilized green leafy vegetables (UGLVs) in bakery items. The searching and the study selection steps were done between February 2025 and March 2025. Only studies published from January 2005 up to January 2025 were considered eligible for the review.

All retrieved records were brought into Rayyan, that web-based systematic review screening platform, for duplicate identification and then study selection sort of thing. After the duplicates were removed, the titles and abstracts were screened against the predefined inclusion and exclusion criteria, and then any potentially eligible studies moved on to full-text assessment. The whole study selection process was done in line with the Preferred Reporting Items for Systematic Reviews and Meta-Analyses (PRISMA) guidelines, more or less.

For the search strategy, different combinations of Medical Subject Headings (MeSH) terms were used to get better search results, you know, for the more relevant studies. The actual search terms include “impact” OR “nutritional use” AND “organoleptic use” AND “nutritional and organoleptic use” AND “impact on nutritional and organoleptic use” AND “underutilized green leafy vegetables” AND “GLV” AND “use of GLV in bakery products.” These terms were then mixed together in various combinations to pinpoint research studies where GLVs were incorporated into bakery formulations, and where the effects on nutritional composition, sensory attributes, and consumer acceptance were evaluated.

To guarantee the reliability and transparency of the review process, the protocol of this study was registered in the Open Science Framework (OSF); DOI: https://doi.org/10.17605/OSF.IO/7YHDZ. This review follows the Preferred Reporting Items for Systematic Reviews and Meta-Analyses (PRISMA) 2020 and PRISMA 2020 for abstract checklists, which can be accessed on the open-access repository: Figshare at https://doi.org/10.6084/m9.figshare.31878151. The table below contains further details on the search strategies, including database-specific queries ( Table 2) and inclusion criteria and exclusion criteria ( Table 3).

**Table 2.** Overview of the search strategy and the count of publications across each electronic database.

Database	Search strategy	No.of publications
PubMed	Review”[TITLE/ABSTRACT]) AND (“Food Application”[TITLE/ABSTRACT]) AND (“Novel Food Application”[TITLE/ABSTRACT])	1706
Scopus	(“Underutilized green leafy vegetables”) AND TITLE-ABS-KEY (“GLV”) AND TITLE-ABS-KEY (“Impact on nutritional and organoleptic use”) AND TITLE-ABS-KEY (“Use of GLV in bakery products”) OR TITLE-ABS-KEY (“Food Application”) AND TITLE-ABS-KEY (“Novel Food Application”)	580
Web of Science	TS = (“Nutritional use” OR “Organoleptic use” OR “Nutritional and organoleptic use” OR “Impact on nutritional and organoleptic use”) AND TS = (“Underutilized green leafy vegetables” OR “GLV”) AND “Use of GLV in bakery products”) AND (“Novel food application”)	1109
Google Scholar	Allintitle (“Underutilized green leafy vegetables” OR “GLV”) AND (“Impact on nutritional and organoleptic use”) AND (“Use of GLV in bakery products”) AND (“Food application”) AND (“Novel food application”) AND (“Nutritional enhancement”) AND (“Sensory Acceptability) AND (“Fortification”)	5024

**Table 3.** Criteria for inclusion and exclusion of studies.

Inclusion criteria	Exclusion criteria
Review articles, meta-analyses, and systematic reviews relevant to the topic.	Studies lacking experimental data or proper control groups.
Studies that evaluate the nutritional and organoleptic properties of underutilized green leafy vegetables (GLVs).	Research not specifically focused on underutilized GLVs in bakery products.
Studies examining the impact of GLVs on the nutritional profile, sensory attributes (color, texture, taste, aroma), and functional properties of bakery products.	Studies assessing consumer acceptability, sensory evaluation scores, and shelf-life improvement.
Reports on changes in nutritional composition (protein, fiber, vitamins, minerals, bioactive compounds) in bakery products with GLVs.	Studies not reporting nutritional analysis or sensory evaluation results.
Last ten years articles were included for conducting systematic review.	Articles published before ten years are excluded.

## Results

**Figure 1.**
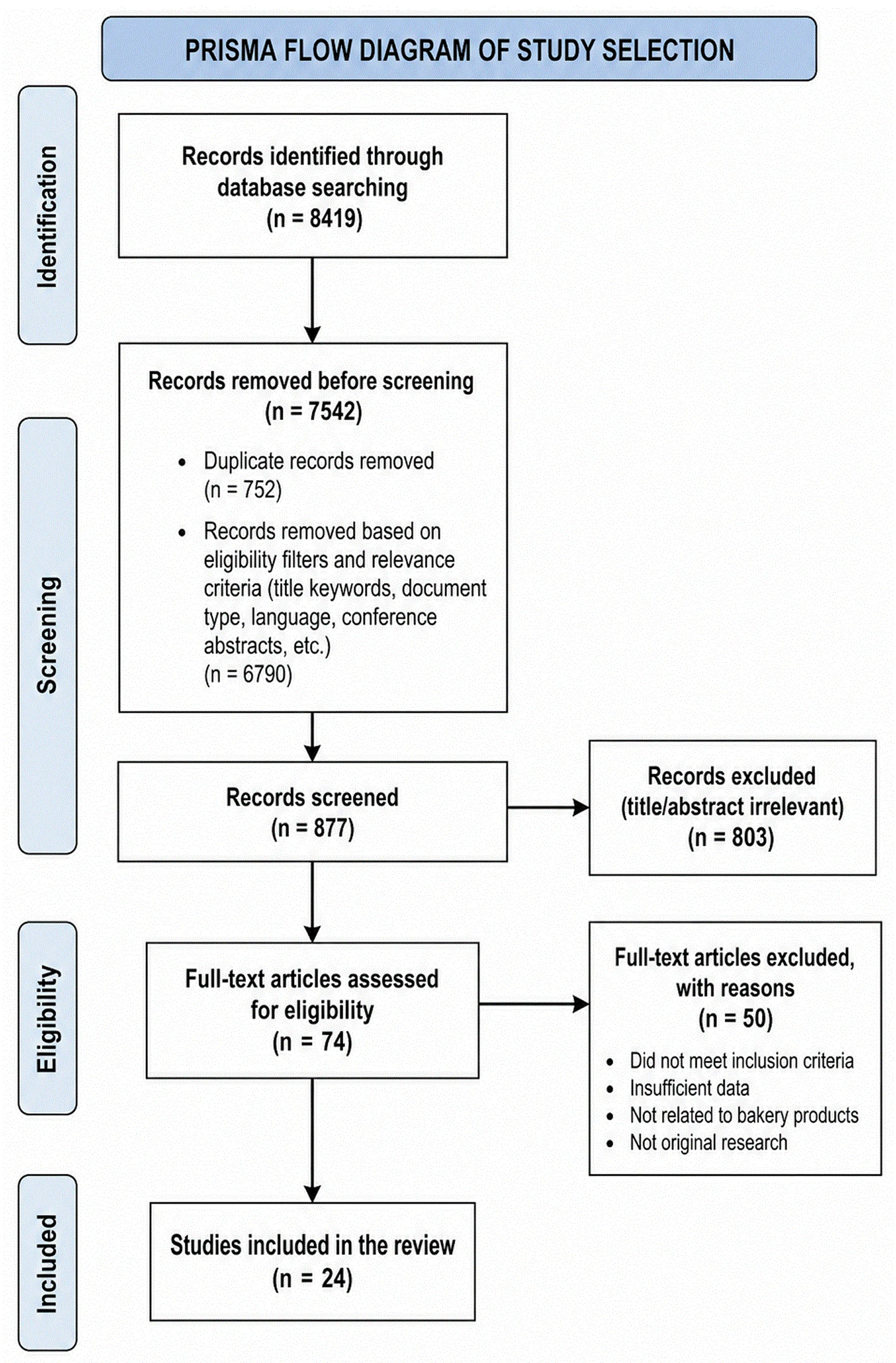
PRISMA 2020 flow diagram illustrating the review process. (n: total count).

A rather comprehensive search across four electronic databases (PubMed, Scopus, Web of Science and Google Scholar) found in total 8,419 records. After removing duplicates (n = 752), another 6,790 records were filtered out before any screening stage, using the already set eligibility and relevance requirements. These exclusions covered a bunch of things like non-bakery food uses, studies that did not involve green leafy vegetables, plus conference abstracts, editorials, review articles, book chapters, non-English publications, and papers that did not really match the goals of this review. In the end, 877 records went through the title and abstract screening. From that group, 803 records were ruled out because they did not satisfy the inclusion criteria. Later on, 74 full-text articles were checked for eligibility and 50 of them were excluded due to insufficient experimental evidence, missing or weak outcome measures, no proper control comparisons, or simply being unrelated to the nutritional and organoleptic potential of underutilized green leafy vegetables (UGLVs) in bakery goods. Ultimately, 24 studies were judged suitable and included in the qualitative synthesis of this review ( Gupta et al., 2005; Sarkar et al., 2023; Okunlola et al., 2023; Yadav & Singh, 2024; Bhavithra et al., 2019; Marappan et al., 2024; Pandiyaraj et al., 2024; Joshi & Mathur, 2015; Coppock et al., 1952). The PRISMA 2020 flow diagram was applied to show how identification, screening, eligibility assessment, and final inclusion unfolded ( Figure 1).

### Attributes of included studies

The selection criteria are summarized in Table 4. Of the 24 selected studies, 15 were published in the last ten years and five were studies examining the impact of GLVs on the nutritional profile and sensory attributes, and four evaluated the nutritional and organoleptic properties of underutilized green leafy vegetables. The studies were conducted in several countries, including India, China, the USA, and other European countries, and were published between 2005 and 2025 ( Gupta et al., 2005; Sarkar et al., 2023).

**Table 4.** Comparative Features of Underutilized Green Leafy Vegetable (UGLV) Incorporation in Bakery Products.

S. No.	Context	Comparative Summary of Findings	First Author(s)	Year
1	Underutilized Green Leafy Vegetables (UGLVs)	Underutilized GLVs are nutrient-dense yet largely neglected, often regarded as weeds or medicinal plants rather than food crops. Their promotion can improve dietary diversity, nutritional security, agricultural biodiversity, and provide economic opportunities for smallholder farmers. Despite their health benefits, limited consumer awareness, inadequate commercial cultivation, and poor market demand continue to restrict their utilization.	Gupta S. et al.; Pandiyaraj et al.	2005; 2024
2	Nutritional Composition and Functional Properties	UGLVs are rich sources of iron, calcium, zinc, potassium, β-carotene, vitamins A, C and E, dietary fibre, phenolic compounds, flavonoids, and antioxidants. Their bioactive constituents contribute to antioxidant, anti-inflammatory, and disease-preventive properties while improving the nutritional profile of fortified foods. However, nutrient composition varies considerably among GLV species and processing methods.	Sarkar T. et al.; Okunlola G.O. et al.; Marappan et al.; Kanwar et al.; R et al.	2022, 2023, 2024
3	Health Benefits and Nutritional Significance	Regular consumption of UGLVs has been associated with a reduced risk of micronutrient deficiencies and chronic diseases due to their high micronutrient density and phytochemical content. Species such as red spinach, amaranth, cauliflower leaves, and carrot leaves provide significant quantities of protein, dietary fibre, vitamins, and minerals that support functional food development and nutritional diversification.	Okunlola G.O. et al.; Sarkar T. et al.; Kanwar et al.; Yadav & Singh	2023; Various; 2024
4	Food Fortification Potential and Bakery Applications	UGLVs have considerable potential as functional food ingredients for bakery products including bread, biscuits, cookies, cakes, crackers, and muffins. Dehydrated or powdered leaf forms are the most commonly used because they improve storage stability and facilitate incorporation into dough. Fortification generally enhances dietary fibre, protein, mineral, vitamin, and antioxidant contents; however, the extent of improvement depends on GLV species, processing methods, bakery formulation, and substitution level.	Yadav S. et al.; Joshi P. et al.; Sarkar T. et al.	2024; 2015; 2022
5	Technological Approaches for GLV Incorporation	Successful incorporation of GLVs into bakery products requires optimization of ingredient substitution, dough formulation, fermentation, drying techniques, and processing conditions. Sourdough fermentation, hydrocolloid incorporation, gluten-free flour substitutes, hydration optimization, and high-pressure processing have been reported to improve dough rheology, texture, product quality, and nutritional retention. Functional ingredients and vegetable by-products further enhance sustainability and product functionality.	Belokurova & Solokhin; Naqash et al.; Menaka et al.; Valsalan et al.; Maria Santamaria et al.; F. Salehi et al.; Ke Qi Lau et al.	2015; 2017; 2023; 2024; Various
6	Quality, Organoleptic and Sensory Characteristics	Sensory quality remains one of the principal challenges limiting commercialization. Incorporation of GLVs may alter dough rheology, loaf volume, crumb texture, colour, flavour, aroma, mouthfeel, and overall acceptability, particularly at higher substitution levels. Gluten-free formulations present additional technological difficulties due to the absence of gluten structure. Consumer acceptance is influenced by flour extraction rate, shortening type, additives, and processing conditions, highlighting the importance of optimizing incorporation levels to balance nutrition with sensory quality.	Alashbayeva et al.; Miguel A. Gallardo et al.; Coppock et al.	Various; 1952
7	Challenges, Sustainability and Future Perspectives	Major barriers to large-scale application include perishability of fresh GLVs, seasonal availability, high moisture content, nutrient losses during processing, dough handling difficulties, and limited consumer awareness. Nevertheless, dehydration technologies, powder preparation, utilization of vegetable by-products, and innovative processing strategies offer opportunities to improve shelf life, reduce food waste, increase sustainability, and develop commercially viable functional bakery products. Future research should focus on standardized processing methods, optimization of incorporation levels, industrial-scale validation, and consumer acceptance studies.	Bhavithra P. et al.; Khatoniar et al.; Aiping et al.; Naumenko N.V. et al.; Kryukova & Kryukov; Gómez et al.; Cano-Lamadrid et al.	2019; 2017; 2018; 2023; 2024; Various

### Attributes of excluded studies

Studies that did not present experimental data were excluded as a criterion by which the merit of the study findings could be judged. Furthermore, studies lacking proper control groups were excluded to allow reliable comparisons between test and control samples. Participants who considered underutilized green leafy vegetables but had no specific inclinations about their application in bakery products were also excluded. Furthermore, studies that did not include any nutritional analysis or sensory evaluation were excluded, as these were essential to show the effect of GLVs on bakery formulations. Articles published before ten years were excluded. There were 50 studies rejected on these grounds.

### Advancement in nutrition bioactive component

The addition of GLV to baked goods significantly increased the levels of dietary fiber, vitamins (A, C, E, K, and B-complex), minerals (iron, calcium, and zinc), and bioactive compounds (polyphenols, flavonoids, and carotenoids). Some studies reported that adding GLV to bakery products improved their antioxidant properties, thus imparting additional functionalities to functional foods. For example, according to Sarkar et al. (2023), GLV-added bakery products had 30-50% more total phenolic content and antioxidant activity than their plain equivalents ( Sarkar et al., 2023).

### Assessment of sensory context and consumer perception

Although there have been improvements in nutrition, the vital sensory parameters have become attributes such as bitterness, color, and texture, which are all considered critical determinants of acceptability by consumers. The overall mean acceptability scores ranged between 6.5 and 8.2 on a 10-point hedonic scale in the six studies. In response to all these problems, dehydration and fermentation were recommended by diverse studies as ways to reduce undesirable sensory characteristics, and this was supported by Coppock et al. (1952) and Naqash et al. (2017).

### Challenges of technical feasibility and processing

The major technological issues faced by GLVs during incorporation into bakery formulations include changes in dough rheology, texture, and shelf-life stability. The addition of GLV powders influenced moisture absorption behavior, thus affecting dough elasticity and crumb structure. Fermentation and oxidative treatments help improve texture and palatability, and can be used in combination with complete drying processes such as hot air and freeze drying, which help preserve bioactive compounds and valuable sensory attributes. These technical observations have been previously reported by Belokurova et al. (2015) and Menaka et al. (2024).

### Study quality evaluation and limitations

Quality assessment based on the Newcastle-Ottawa Scale (NOS) showed that the included studies had scores ranging between 6 and 9, most being medium to high grade. However, heterogeneity in study designs, GLV types, processing methods, and analytic techniques precluded a feasible quantitative synthesis or meta-analysis.

In summary, according to 10 out of 24 studies, the incorporation of GLVs improved the nutritional and functional properties of bakery products, while 14 did not show a significant difference. Owing to the different methodologies and experimental protocols, complete quantitative synthesis is not possible.

## Discussion

Integrating green leafy vegetables (GLVs) into baked goods is a promising strategy to enhance the nutritional value and sustainability of processed foods. Most GLVs have good nutrient compositions, but they remain underutilized because of perishability, sensory difficulties, and lack of consumer knowledge. This discussion will explore facilitation of the incorporation of GLVs into baked products along with the technological, nutritional, and functional aspects of GLVs in bakery products and the challenges and opportunities that arise with them. Advanced technologies, such as ingredient substitution and process optimization, will make GLV incorporation healthy and viable while maintaining the structural integrity of the preferred bakery product. In addition, new applications concerning largely ignored GLVs and traditional grains may resolve sustainability issues in the development of new products. The functional application of these ingredients provides antioxidant capacity and improved product stability. However, emerging barriers to perishability, formulation, and market acceptance should be addressed using innovative processing technologies and consumer education.

The Table 4 present the broad features of GLV incorporation in bakery products, including technological, health, and possible obstacles in upscaling applications. Technical challenges include dough rheology, texture, and shelf-life stability issues. Fermentation and drying processes can improve the texture and maintain the bioactive compounds.

Baked products can be complemented with green leafy vegetables (GLVs) to enhance nutrition and environmental sustainability. GLVs are used sparingly because of their perishability, lack of sensory appeal, and poor consumer awareness. Modern technologies will allow the incorporation of GLVs into products while maintaining their original attributes. Tackling perishability and market acceptability is vital for successful product development.

### Quantitative synthesis of nutritional and sensory outcomes

The included studies consistently reported improvements in dietary fiber, mineral content, antioxidant activity, and sensory acceptability following the incorporation of underutilized green leafy vegetables into bakery products. However, considerable heterogeneity in study design, formulation levels, outcome measures, and reporting formats prevented the generation of a standardized quantitative comparison across studies.

### Industrial and commercial implications of UGLV-based bakery products

The incorporation of underutilized green leafy vegetables (UGLVs) into bakery products presents significant opportunities for the development of functional and value-added foods. Evidence from the studies included in this review demonstrates that UGLVs and vegetable-derived ingredients can be successfully incorporated into a variety of bakery products, including bread, biscuits, cookies, crackers, muffins, and gluten-free formulations. Such incorporation has been associated with improvements in nutritional quality and enhancement of functional properties, thereby contributing to the development of healthier bakery products ( Joshi et al., 2015; Yadav & Singh, 2024; Sarkar et al., 2023). Increasing consumer demand for nutrient-dense and health-promoting foods has further stimulated interest in the utilization of UGLVs as novel ingredients in commercial bakery applications. Among the various forms of incorporation, dehydrated leaf powders are considered particularly suitable for industrial use due to their improved shelf stability, ease of handling, formulation consistency, and standardized incorporation levels when compared with fresh leafy materials ( Joshi et al., 2015; Sarkar et al., 2023).

Shelf-life remains a critical factor influencing the commercial viability of UGLV-enriched bakery products. Fresh green leafy vegetables possess high moisture content, making them susceptible to microbial spoilage and rapid post-harvest deterioration. Consequently, processing techniques such as hot-air drying, freeze-drying, and powder production have been widely explored to enhance storage stability while preserving essential nutrients and bioactive compounds. The utilization of dehydrated leaf powders not only extends ingredient shelf-life but also facilitates year-round availability and incorporation into bakery manufacturing processes, thereby improving their suitability for large-scale commercial production ( Sarkar et al., 2023; Yadav & Singh, 2024).

From a scalability perspective, UGLVs offer a number of good points, like low production costs, making use of underexploited agricultural resources, improving agricultural diversity, and the chance to support sustainable food systems ( Pandiyaraj et al., 2024; Sarkar et al., 2023). Still, when you try to go large scale, commercialization gets tricky, for example seasonal availability, variability in nutrient composition, absence of standardized processing techniques, supply-chain limitations, and consumer acceptance concerns related to color flavour, and texture changes in fortified bakery items ( Bhavithra et al., 2019; Naumenko et al., 2024).

In the Indian context, bringing UGLV-fortified bakery products into the market has to fit under the Food Safety and Standards Act, 2006 and the connected regulations that are released by the Food Safety and Standards Authority of India (FSSAI). Nutritional claims, health claims, ingredient disclosures, labeling requirements, and fortification specific clauses have to follow the FSSAI rules. This kind of regulatory alignment is not just paperwork, it is needed to make sure product safety, consumer trust, and overall market uptake stay on track.

The market outlook for UGLV-based bakery products looks encouraging, especially because the functional foods space and health-oriented bakery segment are both expanding quickly. Consumers are getting more aware about nutrition, sustainability, clean-label ingredients, and plant-based options, so there is room to commercialize bakery products that are enriched with nutrient-dense underutilized vegetables. As a result, UGLV-enriched bakery products could become a useful niche in the developing functional food arena while also supporting food security agricultural sustainability and diet diversification ( Sarkar et al., 2023; Pandiyaraj et al., 2024).

### Strengths and limitations

This systematic review was conducted according to the PRISMA guidelines. It followed comprehensive and systematic procedures for the selection and analysis of the literature. Research from four major databases (PubMed, Scopus, Web of Science, and Google Scholar) was included in this study, regardless of the language. This negates the possibility of a publication bias. This synthesis evaluates the nutritional and sensory aspects of the improvements brought by incorporating underutilized green leafy vegetables into baked products, thereby functioning as functional foods. The findings will also contribute to the emerging field of sustainable nutrition by positing underutilized GLVs as contributors to greater dietary diversity.

However, this review has some limitations. Most of the studies included were either observational or experimental, making it difficult to establish a direct causation between GLV incorporation and direct health outcomes. Diverse designs, processing methods, and analytic techniques did not permit a meta-analysis in this study. In addition, biases could arise from differences in sensory evaluation methods and consumer acceptability assessments, as these are mainly self-reported. However, it does not investigate the long-term stability or commercial acceptability of GLV-laden bakery products. Future studies must standardize the formulas and employ randomized controlled trials for functional and sensory validation in real-life implementation contexts.

## Conclusions

A systematic review of the effects of underutilized green leafy vegetables (GLVs) in bakery products has drawn several striking conclusions. The nutritional benefits of adding GLVs to baked products include the introduction of vitamins and minerals into baked foods, thereby augmenting dietary diversity and nutritional intake.

GLVs not only improve nutritional value but also contain bioactive compounds that provide health benefits, such as glycemic control and antioxidant effects. Thus, GLVs can be considered potential functional foods that are beneficial for health.

Consumer Sensory Challenges: According to the review, nutritional benefits aside, sensory acceptance continues to be a barrier to consumption. Bitterness and texture can both play a role in consumer acceptance. In addition, the threshold of inclusion of GLVs varies according to the specific GLV and processing methods.

**Technological Solutions:** The review suggests the adoption of technological methods, such as drying and fermentation, to address sensory issues. These techniques will help eliminate unwarranted sensory effects, making the GLV bakery more palatable.

**Need for Standardization:** As observed in this review, future research should be directed toward formulating standardized and randomized controlled trials. In this way, functional and sensory properties can be confirmed in a real-life context, ensuring that consumers are catered to and benefits are maximized.

**Gaps in Knowledge:** The review shows that research shows a wide gap in existing literature, especially regarding studies geared towards establishing direct causa and understanding the activity and contribution of individual GLVs in humans.

## Data Availability

**Figshare.** Classification of Underutilized Green Leafy Vegetables. https://doi.org/10.6084/m9.figshare.33171950. The above file contains the underlying data: Classification of Underutilized Green Leafy Vegetables Included in the Review. (It includes the common names, scientific names, families, regions of traditional use, and reasons for UGLV classification). **Figshare**. Data Extraction Table. https://doi.org/10.6084/m9.figshare.31970022 ( Santhanam et al., 2026a). Above file contains the following underlying data: Data extraction table. (The screened articles will be organised in the mentioned data sheet.) **Figshare**. Search Strategy. https://doi.org/10.6084/m9.figshare.31970049 ( Santhanam et al., 2026b). Above file contains the following underlying data: Research study design (The above document includes the study design used for the systematic literature review.) **Open Science Framework (OSF):** Impact of Nutritional and Organoleptic Use of Underutilized Green Leafy Vegetables in Bakery Products: A Systematic Review of Novel Food Applications; DOI: https://doi.org/10.17605/OSF.IO/7YHDZ ( Santhanam et al., 2026c). This project contains the following extended data: OSF project files (Systematic review protocol and related documentation). **Figshare**. Data synthesis table. https://doi.org/10.6084/m9.figshare.31970184 ( Santhanam et al., 2026d). This file contains the extended data table of the synthesised articles, which were finalised after screening for eligibility criteria. **Figshare**: PRISMA CHECKLIST AND PRISMA ABSTRACT CHECKLIST; DOI: https://doi.org/10.6084/m9.figshare.31878151 ( Santhanam et al., 2026e). This project contains the following reporting guidelines: PRISMA checklist.pdf (Checklist followed for systematic review reporting). Study data can be accessed according to the terms of the Creative Commons Attribution 4.0 International License (CC-BY 4.0).
