# Supplementary material for: Impact of Nutritional and Organoleptic Use of Underutilized Green Leafy Vegetables in Bakery Products: A Systematic Review of Novel Food Applications
Source: F1000Res. 2026 Aug 26;15:732. Originally published 2026 May 14. [Version 2] doi: 10.12688/f1000research.180136.2 (PMC13241839; doi:10.12688/f1000research.180136.2)
Supplement: Peer review history

# Reviewer report: Reviewer response for version 2

**DOI:** 10.5256/f1000research.208059.r514757

**Published:** 2026-09-10

**Authors:** Esther Lydia D

**Affiliations:** 1 Loyola College, Chennai, Tamil Nadu, India

The authors have included the recommended inputs, and the article is updated.

Are the rationale for, and objectives of, the Systematic Review clearly stated?

Yes

Is the statistical analysis and its interpretation appropriate?

Not applicable

If this is a Living Systematic Review, is the ‘living’ method appropriate and is the search schedule clearly defined and justified? (‘Living Systematic Review’ or a variation of this term should be included in the title.)

Not applicable

Are sufficient details of the methods and analysis provided to allow replication by others?

Yes

Are the conclusions drawn adequately supported by the results presented in the review?

Yes

Reviewer Expertise:

Functional Foods, Food Processing, Nutrition

I confirm that I have read this submission and believe that I have an appropriate level of expertise to confirm that it is of an acceptable scientific standard.

# Reviewer report: Reviewer response for version 1

**DOI:** 10.5256/f1000research.198722.r485439

**Published:** 2026-06-05

**Authors:** Madhusmita Dishri

**Affiliations:** 1 Vellore Institute of Technology, Vellore, Tamil Nadu, India

The review entitled ‘Impact of nutritional and organoleptic use of underutilized green leafy vegetables in Bakery products: A systematic review of Novel Food applications” addressed the role of underutilized green leafy vegetables on nutritional and sensory properties of bakery products.

1. Researchers studying functional food development and food fortification will find this paper to be a helpful starting point, especially when considering South Asian dietary patterns.
2. This study address a real gap which says GLVs are nutritionally rich but systematically understudied in processed food applications with focus on sensory/organoleptic results and nutritional fortification is practically beneficial.
3. Provides transparency and reproducibility by adhering to PRISMA 2020 requirements with a pre-registered procedure and searched databases that reduce publication bias, demonstrated inclusion/exclusion criteria and quality assessment with  Newcastle-Ottawa scale.
4. However, before it can be regarded as a robust systematic review, a number of methodological issues must be fixed because the conclusions in its current state outweigh the evidence which indicated many studies demonstrated no significant difference.
5. The idea that sensory acceptability is only difficult to "address" is an understatement; bitterness and texture are well-established obstacles that have prevented commercialisation for decades.
6. Table 3 contains a verbatim duplicate: the content descriptions of entries 4 ("Food Fortification Potential") and 5 ("Challenges and Future Directions") are identical, indicating a copy-paste error. Further, it lacks meaningful comparison of findings.
7. The introduction is short, more like narrative instead of summarising the research gaps in the literature.
8. Gap identification is not explained properly, and there is insufficient data on the trade-offs between nutrition and taste when GLVs are added to baked foods.

**Suggestion**

- Elaborate rising concern of micronutrient deficiencies and the importance of nutritional diversification
- Clear definition of GLVs with their geographical distribution, biodiversity and its nutritional significance, wide consumption and global production of Bakery products, role of functional fortification
- Taxonomy of few economically important GLVs species with their botanical description
- Comparative tables of proximate analysis micronutrient ,phytochemical and bioactive compound ,antinutritional factors is lacking
- Details of pre-processing forms used in baking like  dried , powdered, blanched, fresh and its effect in nutrition retention
- Include nutritional impact in Bakery products such as breads, biscuits and cookies, cakes and muffins, crackers and extruded snacks with respects to micronutrients, protein,  effect of baking conditions; temperature and time on micronutrients, glycaemic index, vitamin retention, high temperature effect,  bioavailability considerations,  overall table demonstrating GLV, Bakery product,  key nutritional changes etc .
- Outline the organoleptic and sensory properties: colour and appearance, texture and rheology, flavour and aroma, taste and mouth feel, overall acceptability
- Methodological comparison across reviewed studies in summary table need to be added
- Address the technological and processing. consideration
- Elaborate on the discussion stating the reason for the non-significant difference in types of green leafy vegetables, different processing methods and product types. Expand consumer acceptability and commercial feasibility.

Are the rationale for, and objectives of, the Systematic Review clearly stated?

Yes

Is the statistical analysis and its interpretation appropriate?

Yes

If this is a Living Systematic Review, is the ‘living’ method appropriate and is the search schedule clearly defined and justified? (‘Living Systematic Review’ or a variation of this term should be included in the title.)

Not applicable

Are sufficient details of the methods and analysis provided to allow replication by others?

Partly

Are the conclusions drawn adequately supported by the results presented in the review?

Partly

Reviewer Expertise:

Vegetable Science (Horticulture)

I confirm that I have read this submission and believe that I have an appropriate level of expertise to confirm that it is of an acceptable scientific standard, however I have significant reservations, as outlined above.

# Response

**Published:** 2026-08-05

**Authors:** Madhusudhanan Santhanam

**Affiliations:** WGSHA, Manipal Academy of Higher Education, Manipal, Karnataka, India

**Reviewer Comment 1-5:**

 Researchers studying functional food development and food fortification will find this paper to be a helpful starting point, especially when considering South Asian dietary patterns.

 This study address a real gap which says GLVs are nutritionally rich but systematically understudied in processed food applications with focus on sensory/organoleptic results and nutritional fortification is practically beneficial.

 Provides transparency and reproducibility by adhering to PRISMA 2020 requirements with a pre-registered procedure and searched databases that reduce publication bias, demonstrated inclusion/exclusion criteria and quality assessment with  Newcastle-Ottawa scale.

 However, before it can be regarded as a robust systematic review, a number of methodological issues must be fixed because the conclusions in its current state outweigh the evidence which indicated many studies demonstrated no significant difference.

 The idea that sensory acceptability is only difficult to "address" is an understatement; bitterness and texture are well-established obstacles that have prevented commercialisation for decades.

**Response: **We sincerely thank the reviewer for the encouraging and constructive comments regarding the relevance, novelty, and methodological transparency of our systematic review. We appreciate the recognition of the study's contribution to functional food research and food fortification, particularly in the context of South Asian dietary patterns. We have carefully considered the reviewer's valuable suggestions and have revised the manuscript to strengthen the methodological interpretation, provide a more balanced discussion of the evidence, and emphasize the sensory and commercialization challenges associated with the incorporation of underutilized green leafy vegetables (UGLVs) into bakery products.

**Reviewer Comment 6:**

 Table 3 contains a verbatim duplicate: the content descriptions of entries 4 ("Food Fortification Potential") and 5 ("Challenges and Future Directions") are identical, indicating a copy-paste error. Further, it lacks meaningful comparison of findings.

**Response:** Thank you for identifying this issue. The duplicated content has been removed, and the previous Tables 4 and 5 have been consolidated into a single comprehensive table. The revised table provides a comparative synthesis of the literature by summarizing the nutritional, technological, sensory, and application-related findings, thereby improving clarity and avoiding repetition.

**Reviewer Comment 7:**

 The introduction is short, more like narrative instead of summarising the research gaps in the literature.

**Response:** We appreciate the reviewer's valuable suggestion. The Introduction has been substantially expanded by incorporating information on micronutrient deficiencies, hidden hunger, nutritional diversification, functional foods, and the significance of underutilized green leafy vegetables. In addition, the background has been strengthened to provide a clearer rationale for the review.

**Reviewer Comment 8:**

 Gap identification is not explained properly, and there is insufficient data on the trade-offs between nutrition and taste when GLVs are added to baked foods.

**Response:** Thank you for this constructive comment. A dedicated **Research Gap** section has been added to the Introduction to clearly identify the existing gaps in the literature. Furthermore, the Discussion has been expanded to better describe the trade-offs between nutritional enhancement and sensory acceptability, including the influence of GLV type, incorporation level, and processing methods on consumer acceptance.

# Reviewer report: Reviewer response for version 1

**DOI:** 10.5256/f1000research.198722.r485440

**Published:** 2026-06-04

**Authors:** Esther Lydia D

**Affiliations:** 1 Loyola College, Chennai, Tamil Nadu, India

The study on impact of nutritional and organoleptic use of underutilized GLV in bakery products is well written and the paper follows a sound systemic review framework. However, I am suggesting minor revisions to strengthen the quality of the paper. 

 1. The manuscript lacks a clear operational definition of UGLV. The authors should establish explicit criteria for classify UGLV and provide the list with their English names and scientific names.

 2. The methodology lacks search strings and dates/years used for the screening procedure. 

 3. The paper only presented qualitative synthesis of findings. Quantitative summaries of data on fiber, minerals, antioxdant values, sensory scores need to be incorporated. This will allow the readers to have better understand the magnitude of benefits of UGLV.

 4. The manuscript states 8419 records were identified, 752 duplicates and 877 articles underwent screening. These number appears inconsistently mathematically. Need clarification on records removed for other reason. 

 5. The discussion need to elaborate on the industrial applications as limited data has been provided on scalability, product development, shelf-life, FSSAI regulations involved and market value. Discussion commercialization pros and cons would give this paper more value.

Are the rationale for, and objectives of, the Systematic Review clearly stated?

Yes

Is the statistical analysis and its interpretation appropriate?

Not applicable

If this is a Living Systematic Review, is the ‘living’ method appropriate and is the search schedule clearly defined and justified? (‘Living Systematic Review’ or a variation of this term should be included in the title.)

Not applicable

Are sufficient details of the methods and analysis provided to allow replication by others?

Yes

Are the conclusions drawn adequately supported by the results presented in the review?

Yes

Reviewer Expertise:

Functional Foods, Food Processing, Nutrition

I confirm that I have read this submission and believe that I have an appropriate level of expertise to confirm that it is of an acceptable scientific standard, however I have significant reservations, as outlined above.

# Response

**Published:** 2026-08-05

**Authors:** Madhusudhanan Santhanam

**Affiliations:** WGSHA, Manipal Academy of Higher Education, Manipal, Karnataka, India

**Reviewer Comment 1:**

 The manuscript lacks a clear operational definition of UGLV. The authors should establish explicit criteria for classify UGLV and provide the list with their English names and scientific names.

**Response:**

 A new subsection entitled “Operational Definition and Classification of Underutilized Green Leafy Vegetables (UGLVs)” has been added to the Introduction. The subsection provides a clear operational definition of UGLVs and establishes classification criteria based on cultivation status, market presence, geographical distribution, nutritional value, and contribution to food security. Additionally, a new table listing common names, scientific names, families, regions of traditional use, and justification for classification as UGLVs has been included.

**Reviewer Comment 2:**

 The methodology lacks search strings and dates/years used for the screening procedure.

**Response:**

 The Methods section has been revised to include the search period (February–March 2025), publication period considered for eligibility (2015–2025), database-specific search strategies, and details regarding the use of the Rayyan platform for duplicate removal and study screening. The search strategy table has also been expanded.

**Reviewer Comment 3:**

 The paper only presented qualitative synthesis of findings. Quantitative summaries of data on fiber, minerals, antioxidant values, and sensory scores need to be incorporated. This will allow readers to better understand the magnitude of benefits of UGLVs.

**Response:**

 Thank you for this valuable suggestion. We carefully evaluated the possibility of incorporating a quantitative summary of nutritional and sensory outcomes across the included studies. However, substantial heterogeneity was observed in study design, types of underutilized green leafy vegetables evaluated, bakery product formulations, fortification levels, analytical methods, outcome measures, and reporting formats. Consequently, direct quantitative comparison and standardized synthesis of fiber, mineral, antioxidant, and sensory data across studies were not considered methodologically appropriate. To address this concern, a dedicated subsection has been added to the Results and Discussion section highlighting the overall trends reported in the literature. The revised manuscript now states that the included studies consistently demonstrated improvements in dietary fiber, mineral content, antioxidant activity, and sensory acceptability following incorporation of underutilized green leafy vegetables into bakery products, while acknowledging that methodological heterogeneity precluded a standardized quantitative comparison.

**Reviewer Comment 4:**

 The manuscript states 8419 records were identified, 752 duplicates and 877 articles underwent screening. These numbers appear mathematically inconsistent.

**Response:**

 The PRISMA flow diagram and corresponding text have been revised. Additional records excluded before screening based on predefined eligibility and relevance criteria are now explicitly reported, thereby resolving the numerical inconsistency. The revised PRISMA flow diagram provides a transparent account of study identification, screening, eligibility assessment, and inclusion.

**Reviewer Comment 5:**

 The discussion needs to elaborate on industrial applications, scalability, product development, shelf-life, FSSAI regulations involved, and market value.

**Response:**

 A new subsection entitled “Industrial and Commercial Implications of UGLV-Based Bakery Products” has been incorporated into the Discussion section. This subsection discusses bakery product development opportunities, shelf-life considerations, scalability challenges and opportunities, relevant FSSAI regulatory considerations, and the commercial market potential of UGLV-fortified bakery products.

# Reviewer report: Reviewer response for version 1

**DOI:** 10.5256/f1000research.198722.r485444

**Published:** 2026-06-03

**Authors:** Angeline Esther Preethi S

**Affiliations:** 1 RVS College of Arts and Science, Coimbatore, Tamil Nadu, India

**Summary**

 This review article explores the potential of incorporating underutilized green leafy vegetables (UGLV) in bakery products for application as  functional foods.

 1. The selection of articles for this manuscript is done in a systematic manner using scientific databases, Pub Med, Scopus, Google Scholar and web of Science.

 2.The significance of adding the underutilized vegetables in improving the nutrient content, bio active compounds and functional ingredients is clearly described.

 3.The challenges incurred on addition of these underutilized food ingredients on the functional and sensory attributes of bakery products is also clearly documented

 4. The paper envisages to use food processing technologies to develop UGLV incorporated bakery products with better quality parameters 

 The manuscript is well written with systematic review and analysis and suitable for indexing.

Are the rationale for, and objectives of, the Systematic Review clearly stated?

Yes

Is the statistical analysis and its interpretation appropriate?

Yes

If this is a Living Systematic Review, is the ‘living’ method appropriate and is the search schedule clearly defined and justified? (‘Living Systematic Review’ or a variation of this term should be included in the title.)

Not applicable

Are sufficient details of the methods and analysis provided to allow replication by others?

Yes

Are the conclusions drawn adequately supported by the results presented in the review?

Yes

Reviewer Expertise:

Food processing, Community nutrition, New product development

I confirm that I have read this submission and believe that I have an appropriate level of expertise to confirm that it is of an acceptable scientific standard.

# Response

**Published:** 2026-08-05

**Authors:** Madhusudhanan Santhanam

**Affiliations:** WGSHA, Manipal Academy of Higher Education, Manipal, Karnataka, India

**Reviewer Comment 1**

 The selection of articles for this manuscript is done in a systematic manner using scientific databases, PubMed, Scopus, Google Scholar, and Web of Science.

**Response:**

 We sincerely thank the reviewer for appreciating our systematic literature search methodology. We are pleased that the comprehensive selection of scientific databases, including PubMed, Scopus, Google Scholar, and Web of Science, was recognized as appropriate for ensuring a rigorous and comprehensive review of the available literature.

**Reviewer Comment 2**

 The significance of adding the underutilized vegetables in improving the nutrient content, bioactive compounds, and functional ingredients is clearly described.

**Response:**

 We sincerely appreciate the reviewer's positive comments regarding our discussion of the nutritional and functional significance of underutilized green leafy vegetables. We are grateful that the presentation of their contribution to enhancing nutrient composition, bioactive compounds, and functional ingredients was found to be clear and informative.

**Reviewer Comment 3**

 The challenges incurred on addition of these underutilized food ingredients on the functional and sensory attributes of bakery products is also clearly documented.

**Response:**

 We thank the reviewer for recognizing our discussion of the technological, functional, and sensory challenges associated with incorporating underutilized green leafy vegetables into bakery products. We appreciate the encouraging feedback on the clarity and comprehensiveness of this section.

**Reviewer Comment 4**

 The paper envisages to use food processing technologies to develop UGLV incorporated bakery products with better quality parameters.

**Response:**

 We sincerely thank the reviewer for appreciating the future perspective presented in our review. We are pleased that the discussion on the potential application of advanced food processing technologies for improving the quality and acceptability of UGLV-incorporated bakery products was found to be valuable.

**Reviewer Comment 5**

 The manuscript is well written with systematic review and analysis and suitable for indexing.

**Response:**

 We are sincerely grateful for the reviewer's encouraging remarks and positive assessment of our manuscript. We greatly appreciate the recognition of the quality of the systematic review and analysis, and we thank the reviewer for considering the manuscript suitable for indexing.
